# How can pharmacists develop patient-pharmacist communication skills? A realist review protocol

**DOI:** 10.1186/s13643-016-0396-0

**Published:** 2017-01-23

**Authors:** Aisling Kerr, Judith Strawbridge, Caroline Kelleher, Fien Mertens, Peter Pype, Myriam Deveugele, Teresa Pawlikowska

**Affiliations:** 1 School of Pharmacy, Royal College of Surgeons in Ireland (RCSI), Dublin, Ireland; 2 Division of Population Health Sciences, Department of Psychology, RCSI, Dublin, Ireland; 3 Department of Family Medicine and Primary Health Care, Ghent University, Gent, Belgium; 4 Health Professions Education Centre (HPEC), RCSI, Dublin, Ireland

**Keywords:** Communication, Pharmacy education, Pharmacist, Pharmacy student

## Abstract

**Background:**

Good patient-pharmacist communication improves health outcomes. There is, however, room for improving pharmacists’ communication skills. These develop through complex interactions during undergraduate pharmacy education, practice-based learning and continuing professional development. Research is needed to determine how best to approach teaching patient-pharmacist communication.

**Methods:**

The aim of the research is to understand how educational interventions develop patient-pharmacist interpersonal communication skills produce their effects. A realist review approach will be used to synthesise the literature to make sense of the complexities of educational interventions. Our review will iteratively progress through the various stages of clarifying scope, locating existing theories, searching for evidence, appraisal of papers, data extraction and synthesis. A scoping review revealed a number of substantive theories, which will be used to build an initial programme theory. This will be explored through available published evidence, which we will find by searching databases such as Medline, EMBASE, PsychInfo, ERIC, Scopus and Web of Science. Judgements will be made on the relevance and rigour of the retrieved literature and will be taken into consideration during analysis and synthesis. Synthesis, testing and refinement of the theories will describe and explain the links between contexts, mechanisms and outcomes of educational interventions for communication development in pharmacy.

**Discussion:**

The realist review will provide an analysis of what works when, for whom, how and why, for educational interventions for interpersonal patient-pharmacist communication development. We will also explore barriers to successful communications training and acknowledge any limitations. Ultimately, we plan to provide pharmacy educators with evidence for how best to incorporate educational interventions for communications skills development into pharmacy curricula and for life-long learning opportunities for pharmacists.

**Electronic supplementary material:**

The online version of this article (doi:10.1186/s13643-016-0396-0) contains supplementary material, which is available to authorized users.

## Background

Effective communication with patients is essential and improves health outcomes [1]. The World Health Organization (WHO) report entitled “*The role of the pharmacist in the health care system: preparing the future pharmacist*” identified the pharmacist as a “*communicator*” [2]. More recently, the role of the pharmacist has been expanding in a patient-centred way making communication between patients and the community a vital component of daily practice [3]. Good communication skills are needed for complex activities such as conducting medicine reviews, motivating people to adhere to medicines and health promotion [4].Pharmacists must, therefore, adapt their communication to the wide variety of patient needs and achieve patient-centred communication [5, 6].

Pharmacy competency frameworks emphasise communication as a core competency for pharmacists [7–12]. There have, however, been studies about pharmacists’ communication with patients which suggest that these skills are not always applied [5, 13, 14]. Pharmacy communication skills can be improved with education and training [3, 14–16], and education standards highlight the importance of teaching communication [17–19]. What is known is that pharmacy students need appropriate training and education to develop suitable communication skills, as well as the opportunity to practice these in clinical situations [20]. Advanced training is also needed for qualified pharmacists, particularly those involved in providing extended services, as part of life-long learning [21].

While there is international consensus on the importance of patient-pharmacist interpersonal communication, the education and training is not standardised [22]. Different countries have different definitions of what the essential components of effective patient-pharmacist communication entail, different educational systems and different professional careers for pharmacists. These differences have led to inconsistent communication-based learning outcomes and teaching modalities [15, 23]. A study of instruction and assessment of communication skills in the United States of America and Canada found that most schools of pharmacy reported using lectures as the primary mode of teaching and written examinations for assessing communication skills [24]. Learning activities involving simulated patients are employed to teach communication skills in pharmacy, but are still underutilised [3, 16].

Pharmacists’ communication needs to be further developed towards a more patient-centred approach, and more research is needed to establish how best to teach it to ensure a positive impact for pharmacy education on healthcare outcomes [5].

### Objectives and focus of the review

A systematic review of communications training in undergraduate pharmacy education, 1995–2010, concluded that the effectiveness of previously published educational interventions was uncertain as many were described “*out of context*” of other learning activities that took place [3]. Communication development for pharmacists is a complex intervention as it has multiple components which are reliant on people and are highly dependent on the context in which they take place. Realist review is typically used to explore complex interventions. A realist approach is, therefore, particularly useful for this research as there is little understanding of how and why educational interventions for pharmacists lead to particular outcomes. There are currently no other realist reviews in the pharmacist communication space. Other systematic reviews in this area did not explore how the context of the intervention may influence outcome or why the intervention worked. For this reason, we feel it is important to look beyond the intervention itself using realist methodology [3, 16]. The aim is to produce a realist review that explores the context, mechanisms and outcomes regarding educational interventions for communication skills for pharmacy students in undergraduate and post-graduate education and qualified pharmacists. This will offer pharmacy educators evidence from which to choose the most suitable learning activities to use, in what order and combination, for developing pharmacists’ communication skills within their own teaching context.

### Review question

The aim of the research is to understand how educational interventions ﻿to﻿ develop patient-pharmacist interpersonal communication skills produce their effects. The research questions are as follows:

- What types of educational interventions have been used to develop interpersonal pharmacist-patient communication skills?
- What are the mechanisms by which educational interventions to develop interpersonal patient-pharmacist communication skills are believed to result in their intended outcomes?
- What are the important contexts that determine whether the different mechanisms produce their intended outcome?
- In what circumstances are educational interventions most likely to be effective?
- How should communications training be incorporated into pharmacy curricula?

## Methods/design

### Design

**Fig. 1:**
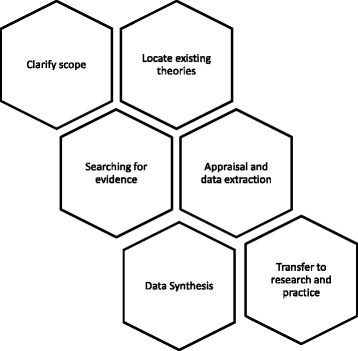
Study design using Pawson’s practical stages of conducting a realist review [25]

The research questions will be addressed using a realist review approach. A realist review asks “*what works, for whom, in what circumstances, in what respects, to what extent and why*?” [25]. Realist research explores the links between contextual factors and the processes or mechanisms these trigger, to explain why and how different outcomes have been achieved [25, 26] This review protocol follows Pawson’s practical stages in conducting realist reviews [25] and is summarised in Fig. 1 in a manner adapted from that reported by Brennan and colleagues [27]. The tessellated diagram illustrates that process is not necessarily linear, and that realist reviews are iterative by nature and movement will be required between stages.

The first step was to clarify the scope of the review, the review questions, nature of communications training and the purpose of the review [25] A scoping review of pharmacy communication skills education helped to clarify this and revealed the potential for further research in this area. This is followed by initial programme theory formulation about how and why particular communication skills interventions might be effective for pharmacists. This iterative process involves exploration of the literature and discussion by the multidisciplinary review team. The team reflects the different perspectives of medical and pharmacy education and behavioural science. We are drawing on research regarding educational interventions for medical communication skills development, as this field is relatively well developed compared with pharmacy. The following initial substantive theories have already been identified as potentially informing how and why communication skills development might occur in pharmacy.

#### Experiential theory

This theory proposes that good communication skills are “*created through the transformation of experience*” [28]. This type of learning is non-formal and is generated through participating in, and observing, experiences in the workplace [29, 30].

#### Structured training theory

This theory posits that communication is not believed to improve through experience alone [31], and that structured, longitudinal training is needed as skills develop with practice [32–34].

#### Reflective theory

This theory ascertains that learners must develop self-awareness, recognise their strengths and weaknesses, deal with uncertainty, be able to adapt to new situations and respond to feedback [35]. Reflection is integral to a deep approach to learning and enhanced professional practice [36].

#### Relational or creative theory

Salmon’s theory of creative communication values learning with real patients to improve relational and emotional dynamics [37].

These theories will be used to inform the initial programme theory development. We will also explore more fully the concept of effective patient-pharmacist communication. We see this as a non-linear process—a dynamic activity between the patient and pharmacist [38, 39]. We are most likely to find that communication development is perceived as the development of skills and behaviours [14, 40–42]. This exploration will also be developed through multidisciplinary discussion, and all aspects of the initial programme theory will be informed by seeking perspectives from wider stakeholders. This realist review protocol has not been registered in any database.

### Searching process

The next step in the process will be to search the literature in order to refine the programme theory of how educational interventions develop interpersonal patient-pharmacist communication [25].

A broad literature search will be undertaken within the following databases: Medline (PubMed), EMBASE, ERIC for educational resources, Web of Science and PsycInfo. Search terms have been developed iteratively (through piloting and refinement) through discussion with the multidisciplinary team and with the assistance of a librarian. Search terms use subject headings, where thesauri exist, in conjunction with free text terms using truncation and appropriate Boolean operators. A combination of MeSH terms and keywords will be used to capture studies of interest. The searches will be designed to ensure focus on the review questions, and to ensure we include pharmacists along all stages of their professional journey from pharmacy students in pre-qualification education (undergraduate or pre-registration or interns during in-service practical training) to pharmacists undertaking post-graduate qualifications and continuing professional development. We also intend to capture any approach to developing interpersonal patient-pharmacist communication skills including interventions and opportunistic learning of communication skills. As this is a realist review, the intervention will not be pre-defined and no restrictions will be imposed on the type of study or interventions. The interventions may include, but are not limited to role play, case-based learning, interactive exercises, improvisational exercises, films, videotapes, audiotapes, self-assessment, peer-assessment, standardised patients, real patients and workplace-based experiential learning.

The searches will be undertaken by a member of the team (AK) and will focus on identifying research published in English from 1996 onwards. The date was chosen to capture 20 years of data, informed by the scoping review and with due regard for the time limit and resources available for completing the review. Keywords will be used and the search string will be translated between databases to ensure the search is refined. The search will be supplemented by hand-searching references of retrieved articles. Further details on the search terms and search strings can be found in the Additional file 1.

### Selection and appraisal of papers

This will be undertaken in three stages: (1) initial screen by title and abstract, (2) full text retrieval and (3) appraisal. Initial screening by title and abstract will be carried out in duplicate by AK and JS. Each title and abstract retrieved from the searches will be reviewed using the inclusion and exclusion criteria to determine suitability. Papers will be excluded if they are not related to a pharmacy context, such as those relating to other healthcare professions, if they are not related to the development of communication skills or if they are not related to interpersonal patient-pharmacist communication. Although there is a large body of literature on communication education in other health professions, particularly medicine, it was decided to restrict to the pharmacy context as pharmacist-patient communication is unique. We will exclude papers that are concerned with developing interprofessional communication skills which, although is of great interest, is outside the scope of the review. If there is any ambiguity, or any discrepancies, these will be discussed between the two reviewers and referred to a third member of the team if necessary (CK).

Each paper that is accepted at this stage will proceed to the full text retrieval. Any study published only in abstract form will be excluded at this stage, as the full paper must be accessible to proceed to full review. AK and JS/CK will review each paper independently and will agree to their conclusions to determine its suitability for inclusion in the review. All articles deemed suitable for inclusion at this stage will proceed to data extraction and appraisal.

Unlike a systematic review for a biomedical intervention which is based on the use of a hierarchy of evidence, realist review rejects a hierarchical approach and uses multiple methods to illuminate the richer picture. A realist review takes a different position on how to judge research quality and aims to examine whether the study is fit for purpose according to relevance and rigour [43]. Relevance is a judgement based on whether the study contributes to theory building and testing. Rigour is a judgement on the credibility of the methods used to generate the data [44]. Relevance will be assessed based on how much information the paper can contribute to programme theory development or refinement, or whether the study is able to inform our understanding of patient-pharmacist communication educational interventions and their respective contexts, mechanisms and outcomes. Studies that meet inclusion criteria will be further assessed for relevance and may be excluded if they do not include any information relevant to programme theory development or refinement. Rigour will be assessed in accordance with the type of evidence, and any limitations of the methods used to generate the data will be taken into consideration during analysis [45]. It is expected that the data obtained will include a mixture of qualitative and quantitative studies.

### Data extraction

Once agreement on suitability for inclusion has been reached, all included articles will be reviewed in duplicate by two of the reviewers (AK/JS/CK/TP). The team will discuss the data extractions and will agree to their conclusions for each paper. In the case of disagreement, this will be resolved through discussion and if no resolution is found the moderator from the group (TP) will review the paper in an effort to reach a consensus view.

Study characteristics of the included papers will be entered separately into a data extraction form, which has been developed by AK in consultation with the team. All full texts of the included papers will be uploaded into NVivo. Relevant text fragments of each paper, relating to contexts, mechanisms, outcomes and themes that contribute to programme theory refinement will be coded. Coding will be a combination of deductive and inductive. A large amount of deductive coding, based on the initial programme theory, will be carried out. Inductive coding is also likely, as it is envisaged that other, unexpected themes and mechanisms and, therefore, codes will appear during the review process.

### Synthesis of extracted evidence and transfer to research and practice

The included studies will be reviewed to identify data which support or refute the initial programme theory or which add any additional issues or information. As this is a realist review, it is expected that the data obtained will be quite heterogeneous and data will be coded in NVivo in order to enable modelling and facilitate the recognition of emergent patterns. In this way, NVivo will be used to sort these patterns and statements into categories and theories to visualise a model of the links between contexts, the mechanisms and outcomes of educational interventions for communications development in pharmacy. It is envisaged that a number of context, mechanism, outcome links will be identified. It is expected that depending on the context, particular mechanisms will lead to certain outcomes and the coded data is expected to explain these links. Each CMO will be presented as a series of contextualized decision points that can be read as a sentence such as ‘in this context, that mechanism generates this outcome’ or in other words “*if A, then B*” or “*in the case of C, D is unlikely to work*” [25]. It is also planned to produce a diagrammatic illustration of the links between context, mechanism and outcomes and how these links programme theory development. The model will be refined based on included data and additional searches if necessary [43].

Using this iterative process, the initial programme theory will be refined based on interrogation and analysis of this data. During the refinement and analysis process, one or more middle-range theories of how and why particular mechanisms generate certain outcomes within certain contexts and a middle-range theoretical explanation of the pattern of CMOs found will be generated. Once data analysis is complete, a programme theory for how educational interventions for interpersonal patient-pharmacist communication improvement will be developed based on an analysis of CMO configurations.

## Discussion

### Potential expected outcomes and implications for education, research and practice

This review aims to explore the complexity of developing patient-pharmacist communication skills from undergraduate education through to life-long learning. Realist methodology will allow us to examine the relationships between possible contexts, mechanisms and outcomes, and the initial programme theory will be refined based on the evidence found. We will prepare the final paper using the RAMESES publication standards for realist review [44] and the PRISMA-P statement (included as Additional file 2). We expect to be able to make recommendations for pharmacy educators who wish to develop interpersonal patient-pharmacist communications educational interventions in pharmacy education. We acknowledge that the work is likely to be limited by the lack of relevant research of high rigour, and we will recognise any limitations in the final synthesis and recommendations. Our ambition is to make recommendations that will include:

1. A summary of educational interventions, which have been used to develop interpersonal patient-pharmacist communication skills.
2. An analysis of what works when, for whom, how and why, for interpersonal patient-pharmacist communications development.
3. An analysis of what does not work in communications training, including barriers to communication education.
4. Guidelines for incorporation of communications training into pharmacy curricula.

### Plans for updating the review and further research

The team plans to maintain and update the bibliography related to the review question. It is intended that a new search will be conducted 5 years after publication using the initial and additional search strategies to inform of the necessity of an update to ensure that the review remains relevant for pharmacy educators in practice.

## Additional files

Additional file 1: How can pharmacists develop patient-pharmacist communication skills—search strings. (DOCX 17 kb)
Additional file 2: PRISMA-P checklist. (DOCX 38 kb)

## Abbreviations

PRISMA-P: Preferred Reporting Items for Systematic review and Meta-Analysis Protocols
RAMESES: Realist And Meta-narrative Evidence Syntheses: Evolving Standards
WHO: World Health Organization

## Glossary

CMO configurations: Outcomes and their associated mechanisms and contexts
Middle-range theories: These are theories that are used to explain the interaction between mechanisms and context to influence outcomes. They are explanations for observed patterns in the data and can be tested and refined against empirical data.
