# Supplementary material for: How can pharmacists develop patient-pharmacist communication skills? A realist review protocol
Source: Syst Rev. 2017 Jan 23;6:14. doi: 10.1186/s13643-016-0396-0 (PMC5260031; doi:10.1186/s13643-016-0396-0)
Supplement: Additional file 1: — How can pharmacists develop patient-pharmacist communication skills—search strings. (DOCX 17 kb) [file 13643_2016_396_MOESM1_ESM.docx]

**Additional File – Primary and secondary search strategy using keywords and Boolean operators**

| **Database** | **Search String** |
| --- | --- |
| PubMed | ("education, pharmacy"[MeSH Terms] AND (("communication"[MeSH Terms] OR "communication"[All Fields]) AND Skills[All Fields])) AND ("Assessment"[Journal] OR "assessment"[All Fields])  "education, pharmacy"[MeSH Terms] AND (("communication"[MeSH Terms] OR "communication"[All Fields]) AND Skills[All Fields]) (("pharmacy"[MeSH Major Topic] OR "pharmacies"[MeSH Major Topic]) AND ("education"[Subheading] OR "education"[All Fields] OR "educational status"[MeSH Terms] OR ("educational"[All Fields] AND "status"[All Fields]) OR "educational status"[All Fields] OR "education"[All Fields] OR "education"[MeSH Terms])) AND ("communication"[MeSH Terms] OR "communication"[All Fields]) ((("pharmacy"[MeSH Terms] OR "pharmacies"[MeSH Terms]) AND "students, pharmacy"[MeSH Terms]) AND ("communication"[MeSH Terms] OR "communication"[All Fields])) AND ("Assessment"[Journal] OR "assessment"[All Fields])  (((Interpersonal) NOT Interprofessional)) AND ((((((((Pharmacist) OR Pharmacy Student) OR Pharmac*) OR Pharmacy) OR Pharmacy Education) OR Pharmacy Education[MeSH Terms])) AND (((communication) OR communicat*) OR Communication[MeSH Terms])) |
| ERIC | abstract: Communication AND pharmacy education OR pharmacy student  Pharmacy AND Patient AND Communication  Pharmacy AND Interpersonal AND Communication |
| EMBASE | Pharmacy education AND communication |
| Web of Science | TOPIC: ((Pharmacy Education) AND (Communication) AND (Student))  **TOPIC:** (Pharmacy Education) *OR* **TOPIC:** (Pharmacy) *OR* **TOPIC:** (Pharmacist) *OR* **TOPIC:** (Pharmacy Student)  **Refined by:** **TOPIC:** (Interpersonal Communication)  **TOPIC:** (Pharmacy) *AND* **TOPIC:** (Interpersonal Communication) *NOT* **TOPIC:** (Interprofessional Communication) |
| PsycInfo | (Interpersonal Communication and (Pharmacy or Pharmacy Student or Pharmacist)).af.   - communication - interpersonal - interpersonal communication - pharmacist - pharmacy - pharmacy student - student   (Interpersonal Communication and Pharmacy).af.   - communication - interpersonal - interpersonal communication - pharmacy |
